# Assessing the Impact of Nationwide Smoking Cessation Interventions among Employed, Middle-Aged Japanese Men, 2005-2010

**DOI:** 10.1371/journal.pone.0155151

**Published:** 2016-05-10

**Authors:** Koji Wada, Yoshiyuki Higuchi, Derek R. Smith

**Affiliations:** 1 International Health Cooperation, National Center for Global Health and Medicine, Tokyo, Japan; 2 Department of Health and Physical Education, Fukuoka University of Education, Fukuoka, Japan; 3 School of Health Sciences, Faculty of Health and Medicine, University of Newcastle, Ourimbah, New South Wales, Australia; Geisel School of Medicine at Dartmouth College, UNITED STATES

## Abstract

**Background:**

A variety of tobacco control interventions have become available in Japan over the past decade, however, the magnitude to which they have impacted on smoking rates may have varied by socioeconomic status such as job content, particularly for middle-aged men who were formerly long-term smokers. We conducted a longitudinal study to investigate the differences between smoking cessation strategies among a national sample of middle-aged Japanese employed men between 2005 and 2010.

**Methods:**

Data was extracted from a previous longitudinal survey of middle-aged and elderly people that had been conducted by the Ministry of Health, Labour and Welfare. In 2005, 16,738 Japanese men aged 50–59 years were recruited and sent a questionnaire in each year of the study. We analyzed data for individuals who reported being current smokers at baseline. Cox’s discrete time proportional hazard regression analysis was used to examine potential associations between smoking cessation and socioeconomic factors.

**Results:**

Of the 6187 employed, male smokers who participated in 2005, 31% subsequently quit smoking during the 5-year follow-up period. Those working in manufacturing, transportation, or security were less likely to have quit smoking than those working in management. Having no marital partner, never having been married, or those experiencing psychological distress were significantly less likely to have quit smoking during this time.

**Conclusions:**

Although almost one-third of middle-aged, male smokers quit their habit between 2005 and 2010; the uptake of this national strategy appears to have been far from uniform across Japanese society. Socioeconomic factors such as occupation, marital status and psychological distress were negatively correlated with quitting, suggesting that these groups should be more aggressively targeted in further interventions.

## Introduction

Although Japan has been known to be a challenging environment for antismoking movements [1], in the past decade a variety of tobacco control reforms have been enacted nationally, particularly those relating to smoking cessation. In 2002, for example, the Health Promotion Law was promulgated and this has been followed by practical measures such as the introduction of nicotine replacement therapy [2] which is covered by the national insurance scheme, thereby reducing out-of-pocket expenses by up to 30% in 2008 [3]. Other measures have also included ratification of the WHO Framework Convention on Tobacco Control by the Japanese government; strengthening the prevention of passive smoking in public places including workplaces by restricting the permitted locations and times for smoking [4]; and increasing taxes on tobacco. The latter move has increased the price of packaged tobacco products from an average of 270 yen (2.3 US dollars; 1 US dollar = 115.9 yen) in 2003 to 410 yen (4.7 US dollars; 1 US dollar = 87.8 yen) in 2010 [5].

While these national strategies have targeted all smokers in Japan, their uptake does not appear to have been uniformly successful, particularly when considered across the demographic spectrum. Various studies have examined the associations between smoking prevalence and socioeconomic status in Japanese people; with investigations of lower income [6], lower education levels [7], poor health habits [8], and mental distress [9–11] having now been reported. Despite this fact, few studies have examined potential associations between smoking cessation uptake and socioeconomic factors (especially regarding occupation)among middle-aged men, who have hitherto shouldered much of the tobacco-related burden in this country (due to the high prevalence of smoking in this demographic) [12, 13]. National datasets collected by the government represent a feasible strategy for examining these issues at a country-level, and have been previously used in Japan for tobacco control research [14].

Other more recent studies have also been conducted in this regard. Since 2005, for example, the Japanese Ministry of Health, Labour, and Welfare (MHLW) has conducted a nationwide Longitudinal Survey of Middle-Aged and Elderly Persons [15], which targets people in their 50s and 60s and focuses on issues related to health and welfare. This survey also captures smoking status of the sampled population over several years, as well as recording socioeconomic factors. Such data offers a unique opportunity to examine the impact of smoking cessation by socioeconomic factors, especially for Japanese men in their 50s, for whom the prevalence of smoking has historically been high. In 2005, for example, the historical prevalence of smoking among a cohort of Japanese men was 82% when they were aged in their 20s (in 1975), 70% when they were in their 30s (1985), 62% in their 40s (1995), and 49% in their 50s (2005) [16]. Viewed in context (over time) and by severity (almost half were still smoking in 2005), this data suggest that middle-aged Japanese men are particularly addicted to nicotine and offer an appropriate target group for tobacco cessation activities. The current study was undertaken therefore, to examine potential associations between smoking cessation and socioeconomic factors such as occupation, across a national sample of middle-aged, Japanese men (50–59 years), between 2005 and 2010.

## Methods

### Participants and survey method

We obtained data from the MHLW Longitudinal Survey of Middle-Aged and Elderly Persons which began in 2005 [15]. The MHLW randomly selected 2515 regions from a total of 5280 census regions used in the Comprehensive Survey of Living Conditions, and the National Health and Nutrition Survey. All individuals aged 50–59 years in 2005 were eligible for inclusion, giving a total of 40,877 men and women. In the first survey wave, a total of 34,240 men and women responded, giving a response rate of 84%. The MHLW then mailed a questionnaire to participants in each follow-up year, to monitor ongoing health outcomes and working conditions. The response rate from 2005 to 2010 ranged from 92% to 97%. We extracted data for 16,738 males who had participated in the study from 2005 to 2010. Data from respondents who reported they were currently smoking and employed in 2005 were used as the baseline, since we were seeking to longitudinally examine their quitting success over time.

### Measurement

#### Outcome (smoking cessation)

Each year of the study, participants answered a question on their smoking status (currently smoking, quit smoking or have never smoked) on the MHLW survey.

#### Covariates

Baseline data for ‘job content’ was used to record participants’ occupation (divided into nine categories regarding the contents of their daily work: management, professional, manufacturing, sales, service, clerk, transportation, agriculture or security). In addition, education level was examined (using the classifications of: graduated from junior high school, high school, vocational college, university or postgraduate study), marital status (using the discrete variables of married, divorced / widowed or never married) and psychological status using the Kessler 6 (K6) scale (classified into 3 categories for the analysis as: 0–4 indicating no distress, 5–12 indicating psychological distress and over 13 indicating serious mental distress, based on the specified cut-off point) [17].

### Statistical analysis

Statistical analyses were based on the incidence rates of smoking cessation during the 6-year follow-up period (2005–2010). Discrete time survival analyses with Cox’s proportional hazard models were used to determine the hazard ratio (HR) of socioeconomic factors with regard to smoking cessation. Participants were censored at the point they first selected the questionnaire option of ‘quit smoking’ or when they were lost to follow-up. First, we conducted univariate analysis and then a multivariate analysis with covariates such as job contents, education, marital status and K6 score. In addition, Kaplan-Meier curves and log-rank tests were used to compare the cumulative incidence of smoking cessation for two occupations and three different levels of education. SPSS Version 20.0 (IBM SPSS, Armonk, NY, USA) and R software was used for the statistical analyses.

### Ethics

The Statistics Act of Japan allows the MHLW to provide de-identified data for research purposes. The first author gained approval from the MHLW to obtain the data for this study and undertake the analysis. All records and information had been previously anonymized and de-identified. Respondents had originally agreed to participate in the Longitudinal Survey of Middle-Aged and Elderly Persons when it was conducted by the MHLW.

## Results

The baseline survey of 2005 comprised 6187 middle-aged, male Japanese smokers who reported being currently employed. Participant characteristics during the baseline survey are displayed in Table 1. The most common occupation was professionals (25%), followed by manufacturing (16%) and management (15%). A total of 31% of participants had quit smoking during the follow-up period. Table 2 indicates HRs for quitting smoking and the their association with socioeconomic factors. By occupation, the highest proportion of smoking cessation during the follow-up period was observed amongst those working in management (34%), with the lowest amongst those employed in the security industry (21%). Individuals working in manufacturing (HR 0.80; 95%CI: 0.68–0.95), transportation (HR 0.79; 95%CI: 0.64–0.97) or security (HR 0.61; 95%CI: 0.42–0.90) were less likely to quit smoking than those working in management. Having no marital partner (HR 0.77; 95%CI: 0.63–0.92), never having been married (HR 0.71; 95%CI: 0.57–0.89), or experiencing psychological distress (as defined by a K6 score of 5–12 = HR 0.86; 95%CI: 0.77–0.97, K6 score over 13 = HR 0.71; 95%CI: 0.51–0.98) were also significantly associated with not having quit smoking during the observation period.

**Table 1:** Participant characteristics at baseline (n = 6187).

	n	(%)
**Job content**		
Management	942	(15)
Professional	1539	(25)
Manufacturing	987	(16)
Service	552	(9)
Sales	528	(9)
Transportation	481	(8)
Clerk	393	(6)
Agriculture	261	(4)
Security	133	(2)
Other	371	(6)
**Completed education**		
Junior high school or high school	4353	(70)
Vocational college	467	(8)
University or postgraduate	1347	(22)
**Marital status**		
Married	5303	(86)
Divorced/widowed	506	(8)
Never married	378	(6)
**K6 score**		
0–4	4660	(75)
5–12	1359	(22)
Over 13	168	(3)
**Smoking cessation during the follow-up period**		
Yes	1921	(31)
No	4266	(69)

**Table 2:** Hazard ratios for quitting smoking and their association with socioeconomic status (n = 6187).

	Cases/Total		Crude model		Adjusted model	
	n	(%)	HR	95%CI	HR	95%CI
**Job contents**						
Management	323/942	(34)	1		1	
Professional	466/1539	(30)	0.86	(0.74–0.99)	0.89	(0.77–1.02)
Manufacturing	268/987	(27)	0.75	(0.64–0.88)	0.80	(0.68–0.95)
Service	183/552	(33)	0.97	(0.81–1.16)	1.03	(0.85–1.23)
Sales	157/528	(30)	0.87	(0.72–1.05)	0.89	(0.73–1.07)
Transportation	127/481	(26)	0.74	(0.60–0.91)	0.79	(0.64–0.97)
Clerk	120/393	(31)	0.83	(0.68–1.03)	0.85	(0.69–1.05)
Agriculture	88/261	(34)	0.92	(0.72–1.16)	0.96	(0.76–1.22)
Security	28/133	(21)	0.59	(0.40–0.86)	0.61	(0.42–0.90)
Others	101/371	(27)	0.79	(0.63–0.99)	0.85	(0.68–1.07)
**Completed education**						
Junior high school or high school	1265/4353	(29)	0.86	(0.77–0.95)	0.90	(0.80–1.00)
Vocational college	153/487	(31)	0.93	(0.77–1.12)	0.97	(0.81–1.17)
University or postgraduate	443/1347	(33)	1.00		1.00	
**Marital status**						
Married	1663/5303	(31)	1		1	
Divorced/widowed	177/506	(23)	0.75	(0.63–0.91)	0.77	(0.63–0.92)
Never married	81/378	(21)	0.68	(0.55–0.85)	0.71	(0.57–0.89)
**K6 score**						
0–4	1460/4660	(31)	1		1	
5–12	364/1359	(27)	0.85	(0.75–0.96)	0.86	(0.77–0.97)
Over 13	37/168	(22)	0.70	(0.50–0.97)	0.71	(0.51–0.98)

HR = hazard ratio, CI = confidence interval

**Fig 1:**
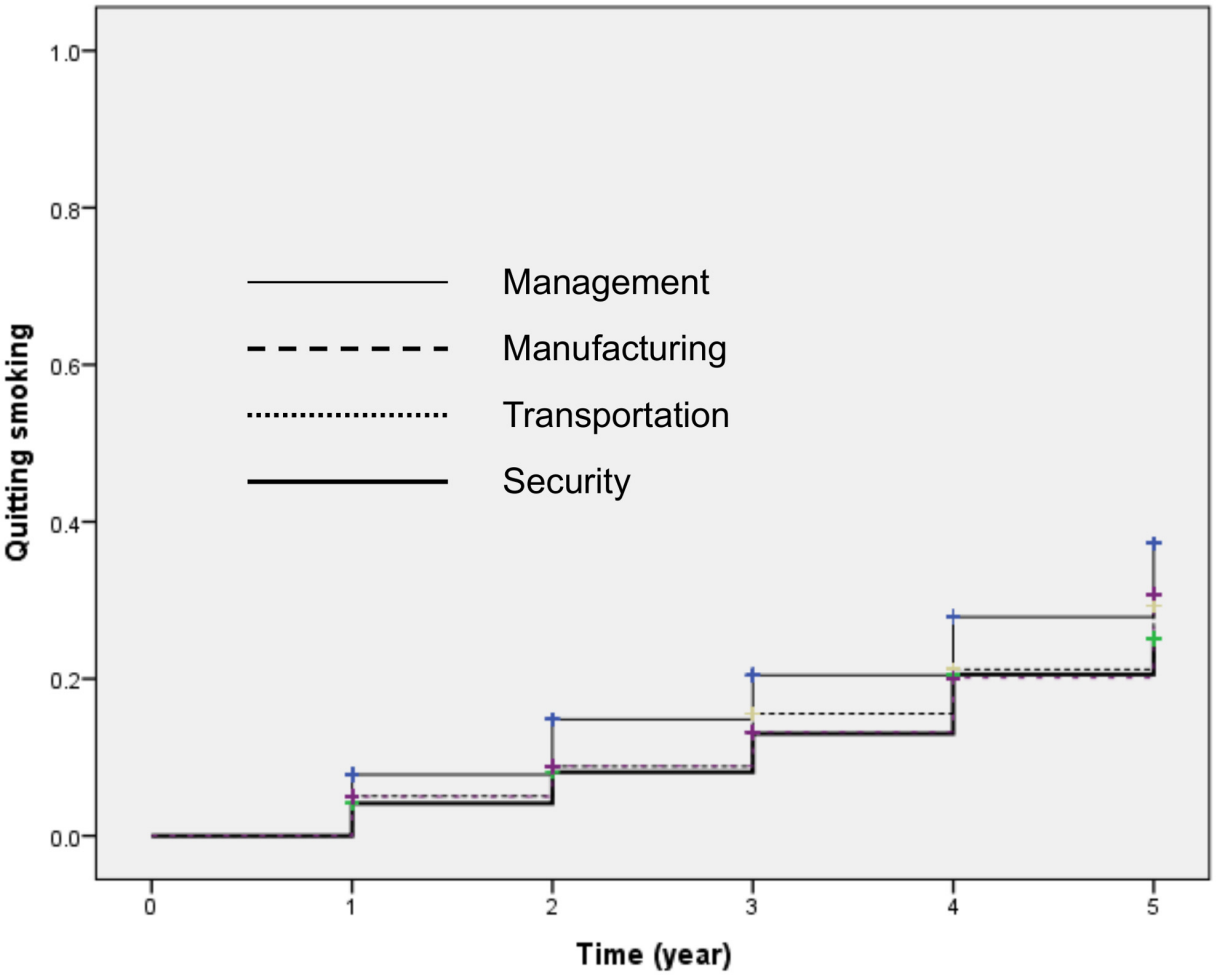
Kaplan-Meier curves for smoking cessation by workers in management, manufacturing, security and transportation (p<0.01, log-rank test).

**Fig 2:**
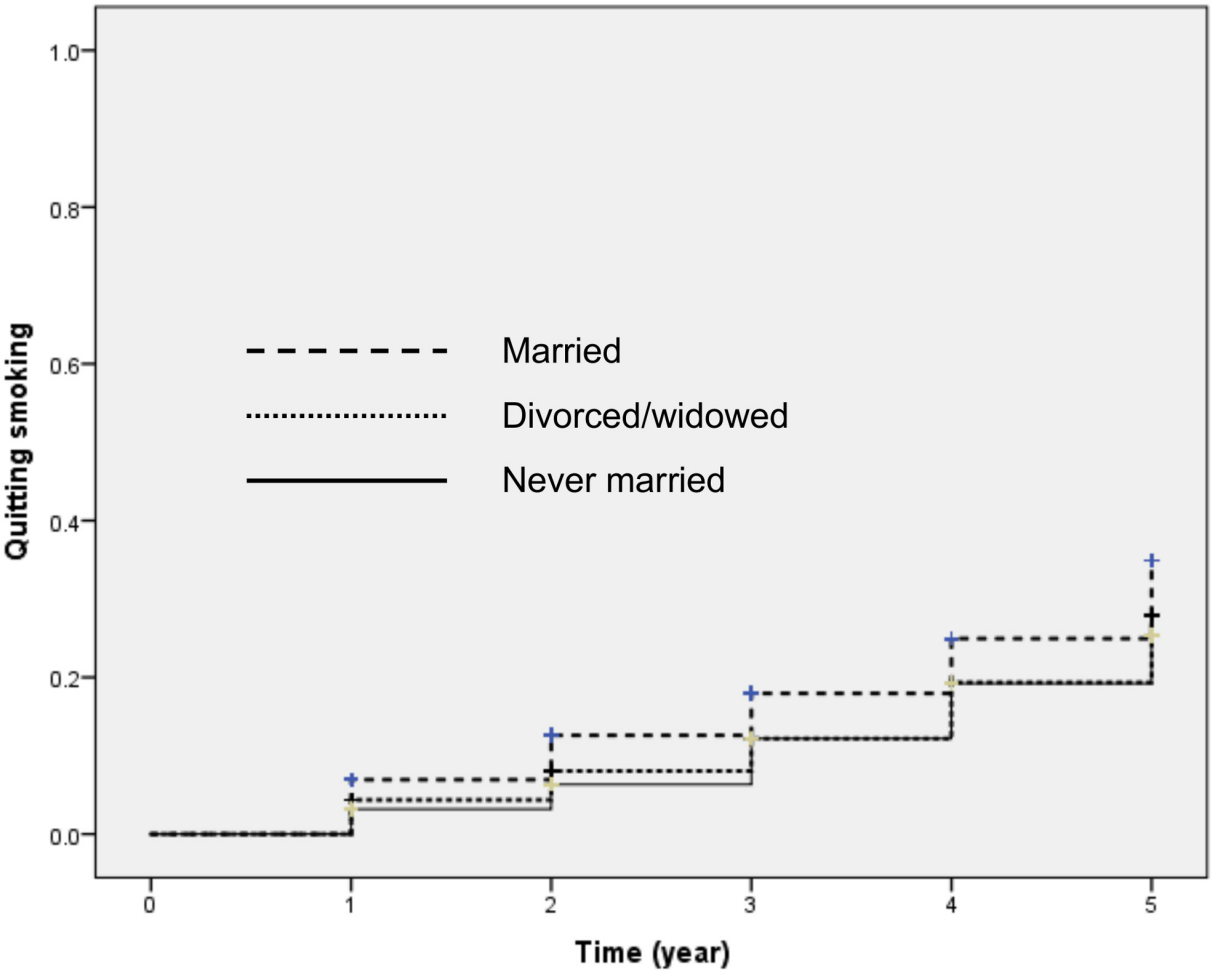
Kaplan-Meier curves for smoking cessation by marital status (p<0.01, log-rank test).

Fig 1 indicates Kaplan-Meier survival estimates for smoking cessation for those in manufacturing, transportation or security; which revealed a significant difference when compared with management (p<0.01; log-rank test). Fig 2 indicates Kaplan-Meier survival estimates for smoking cessation for those with a marital status of divorced / widowed or never married, which indicates a significant difference when compared with participants who were married (p<0.01; log-rank test).

## Discussion

This study examined the impact of national smoking cessation interventions among a cohort of Japanese citizens where a high proportion of the tobacco-related burden historically lies: that being; employed, middle-aged men. Even though all smokers in our investigation had been exposed to the same smoking cessation interventions (tobacco price increases by way of increased taxes, the opportunity to receive nicotine replacement therapy with reduced out-of-pocket expenses, as well as the restriction of smoking in public places including workplaces), the effectiveness of smoking cessation activities was not uniform. Socioeconomic status appeared to play an important role, particularly as seen when comparing participants in management jobs, with those in security, transportation and manufacturing. Having no marital partner and the presence of psychological distress was statistically associated not quitting smoking among our national sample of Japanese men. Our study was unable to determine, however, whether these factors were directly responsible for the lack of tobacco cessation among Japanese men, or rather, indicators of another, unknown variable that could not be elucidated with the current study design. Additional research will be needed therefore, to investigate the role of these correlates. Additional tobacco control interventions that more effectively target individuals within this demographic are also urgently needed in Japan.

Workplace tobacco control, particularly employment-based interventions for smoking cessation offer a novel and as yet underutilized strategy to improve the incidence of smoking cessation [18]. A Cochrane review reported strong evidence for the success of workplace interventions in the form of individual and group counselling, self-help, incentives and pharmacological interventions [19]. However, especially for small or medium enterprises with limited resources, it may be difficult to provide these interventions in workplaces. In Japan, after the enactment of the Health Promotion Law in 2002 that included the requirement to prevent passive smoking in public places; passive smoking in common areas (including workplaces) must now be controlled by segregated smoking areas [20]. Ratifying the WHO Framework Convention on Tobacco Control by the Japanese government [21] also strengthened the fight against passive smoking. This, in turn, seemed to provide momentum for smokers to more seriously consider quitting smoking. In the current study, however, the translation to action was not uniform, with lower cessation rates; for example, observed security workers working outside or alone and transportation workers such as truck drivers working in individual driving spaces. The results of our study suggest, therefore, that more focused initiatives that differentially target Japanese workplaces would be useful in this regard [22].

Socioeconomic status represents another key determinant of smoking status as well as being a predictor for smoking cessation in the current study. Manufacturing, security and transportation jobs that would be expected to employ many individuals from lower socioeconomic strata tended to have lower rates of smoking cessation in the current study. This finding was not unexpected however, as lower social class and lower education levels have been previously identified as risk factors for tobacco use [6, 7]. Interestingly, in the current study, lower education levels (especially junior high school or high school) were only weakly associated with not quitting smoking over time among middle-aged Japanese men. Other research has found that individuals in lower social demographics tend to have a higher risk of relapse after initial smoking cessation [23]. Both results, suggest that intensive support is probably needed for those with lower education levels or those from lower sociodemographic backgrounds. As previously described, the workplace may offer a potential solution for reaching this demographic in a cost-effective manner.

It is important to remember that tobacco smokers in Japan as elsewhere, do not live in a vacuum, and intervention strategies need to consider the wider sphere in which these people live. Marital partners might be able to further encourage smokers to quit smoking; thus helping to prevent premature mortality and simultaneously reduce passive smoking [24, 25]. Although Takagi and colleagues [13] for example, previously reported that spousal smoking status is an important predictor of smoking cessation in men; we did not identify such an association in the current study. Deterioration of mental status has also been highlighted as a risk factor for tobacco use in previous studies [9–11], although it is reasonable to assume that initiating cessation of smoking may be difficult in such populations. Encouragingly however, our findings did reveal that almost one-quarter (22%) of those with serious psychological distress at baseline (indicated by a K6 score over 13) ultimately achieved smoking cessation during the follow-up period. Population approaches for smoking cessation may therefore have some impact for difficult target populations such as this. Given that individuals with mental health problems often find it difficult to access health services, an increased emphasis on smoking cessation by health care workers might offer a potential way forwards. Japanese physicians, of which there are almost 300,000, offer one segment of the healthcare workforce that might be better mobilized to offer tobacco control in their clinical practice [26].

Although our current study represents one of the first of its kind in Japan, it may have incorporated some potential limitations that are worth considering. Some participants who reported quitting smoking during the original follow-up may have later relapsed. Further study needs to address the different reasons for relapse among those who quit smoking during the follow-up periods. Similarly, given that the data were obtained by self-reporting questionnaires, some misclassification and misunderstanding might have also occurred. Furthermore, it could be suggested that the generalizability of results is limited to middle-aged Japanese men. Future national studies of tobacco control in Japan, as elsewhere, would do well to take these issues in to account.

## Conclusions

This study found that although almost one-third of middle-aged, male smokers quit their habit between 2005 and 2010; the uptake of this national strategy was far from uniform across Japanese society. Socioeconomic factors such as occupation, marital status and psychological distress were inversely correlated with quitting, suggesting that these groups should be more aggressively targeted in further interventions.
